# β-Hydroxy-β-Methylbutyrate Supplementation Promotes Antitumor Immunity in an Obesity Responsive Mouse Model of Pancreatic Ductal Adenocarcinoma

**DOI:** 10.3390/cancers13246359

**Published:** 2021-12-18

**Authors:** Michael F. Coleman, Kristyn A. Liu, Alexander J. Pfeil, Suhas K. Etigunta, Xiaohu Tang, Salvador Fabela, Laura M. Lashinger, Zhengrong Cui, Stephen D. Hursting

**Affiliations:** 1 Department of Nutrition, University of North Carolina, Chapel Hill, NC 27599, USA; mcoleman@unc.edu (M.F.C.); pfeilal@live.unc.edu (A.J.P.); suhase@live.unc.edu (S.K.E.); salvador_fabela@unc.edu (S.F.); 2 Department of Nutritional Sciences, University of Texas, Austin, TX 77843, USA; k.liu@utexas.edu (K.A.L.); lalash@austin.utexas.edu (L.M.L.); 3 Department of Biological Sciences, Michigan Technical University, Houghton, MI 49931, USA; xiaohut@mtu.edu; 4 College of Pharmacy, University of Texas, Austin, TX 78712, USA; zhengrong.cui@austin.utexas.edu; 5 Lineberger Comprehensive Cancer Center, University of North Carolina, Chapel Hill, NC 27599, USA

**Keywords:** β-hydroxy-β-methylbutyrate, obesity, pancreatic cancer, immunotherapy

## Abstract

**Simple Summary:**

Pancreatic cancer (PDAC) is a deadly disease, exacerbated by obesity, which lacks effective therapeutic interventions. Most PDAC has a limited response to immune- and chemotherapy. Treating PDAC is made additionally challenging by the rapid emergence of muscle wasting and cachexia, which predict poor response to several therapies. We have found that dietary supplementation with β-hydroxy-β-methylbutyrate promotes immunosurveillance in PDAC tumors and protects muscle. This dietary supplement has the potential to be an important adjuvant in PDAC therapy, opening the doors to immunotherapy response.

**Abstract:**

Pancreatic ductal adenocarcinoma (PDAC) is the fourth leading cause of cancer-related deaths in the United States, and effective therapies for PDAC are currently lacking. Moreover, PDAC is promoted and exacerbated by obesity, while cachexia and sarcopenia are exceptionally common comorbidities that predict both poor survival and treatment response. Managing PDAC with immunotherapies has thus far proven ineffective, partly due to the metabolically hostile tumor microenvironment. β-hydroxy-β-methylbutyrate (HMB), a metabolite of leucine commonly used as a dietary supplement to boost muscle growth and immune function, may be an attractive candidate to augment PDAC therapy. We therefore sought to test the hypothesis that HMB would enhance antitumor immunity while protecting mouse muscle mass. Control and diet-induced obese C57BL/6 male mice bearing subcutaneously injected Panc02 tumors were supplemented with 1% HMB and treated with or without 50 mg/kg gemcitabine (*n* = 15/group). HMB was associated with reduced muscle inflammation and increased muscle fiber size. HMB also reduced tumor growth and promoted antitumor immunity in obese, but not lean, mice, independent of the gemcitabine treatment. Separately, in lean tumor-bearing mice, HMB supplementation promoted an anti-PD1 immunotherapy response (*n* = 15/group). Digital cytometry implicated the decreased abundance of M2-like macrophages in PDAC tumors, an effect that was enhanced by anti-PD1 immunotherapy. We confirmed that HMB augments M1-like macrophage (antitumor) polarization. These preclinical findings suggest that HMB has muscle-sparing and antitumor activities against PDAC in the context of obesity, and that it may sensitize otherwise nonresponsive PDAC to immunotherapy.

## 1. Introduction

Pancreatic ductal adenocarcinoma (PDAC) is the fourth leading cause of cancer-related deaths in the United States. This high mortality rate is exacerbated by the limited efficacy of chemotherapies and immunotherapies [1]. The immunotherapy response is heavily regulated by metabolic features of the tumor microenvironment (TME), such as hypoxia and acidity [2,3]. Thus, interventions to support antitumor immunity are critical if immunotherapy is to become effective in PDAC.

Cancer-associated cachexia, defined by involuntary weight loss and breakdown of adipose and muscle tissue, is present in about 80% of patients with pancreatic cancer and contributes significantly to PDAC mortality [4,5]. Further, sarcopenia and cachexia predict a poor response to immune checkpoint inhibition [6]. Obesity promotes chronic inflammation and immune cell dysfunction [7], and the development and progression of PDAC [8]. A growing proportion of patients with pancreatic cancer are obese at the start of therapy, and many of these individuals have sarcopenic obesity, a condition characterized by the presence of a high fat mass but a low muscle mass. Obesity in the presence of sarcopenia is predictive of morbidity and mortality in patients with pancreatic cancer [9], at least in part because sarcopenic obesity increases the risk of developing cancer cachexia [10]. Preclinical data showed that diet-induced obesity (DIO) promotes PDAC growth [11] and sarcopenia [12] in mice. Obesity is also a critical regulator of the immunotherapy response in some models, promoting an increased response to immune checkpoint blockade [13,14,15,16] and increased toxicity to IL-2 anti-CD40 therapy [17].

β-hydroxy-β-methylbutyrate (HMB), a metabolite of the branched-chain amino acid leucine, is emerging as a strong candidate for increasing muscle mass [18]. HMB supplementation increases muscle growth through activation of the mechanistic target of rapamycin (mTOR) in muscle [19] and attenuates muscle protein degradation through the prevention of nuclear factor-κB (NF-κB) activation [20]. HMB promotes improved function of ex vivo macrophages and monocytes [21,22], but the effects of HMB supplementation on PDAC growth are unknown, particularly in the context of obesity and immunotherapy. Tumor necrosis factor alpha (TNFα) signaling through NF-kB is a pleotropic regulator of the immunotherapy response [23]. NF-κB activation by cytokines such as TNFα is pro-tumorigenic in PDAC, and inhibition of NF-κB reduces PDAC growth in mice [24]. In muscle tissue, NF-κB activation leads to muscle atrophy via increased protein degradation [25]. Inhibition of NF-κB prevents cancer-induced muscle atrophy [26].

Given that muscle wasting is a critical feature of PDAC-associated morbidity and is exacerbated by obesity, new intervention strategies that both suppress PDAC progression and preserve muscle mass in patients with pancreatic cancer and comorbid obesity are urgently needed. Furthermore, interventions that promote a response to immunotherapy while reducing muscle inflammation and can be safely administered may provide a tractable approach to achieve this aim. Herein, we tested the hypothesis that immunomodulation by HMB supplementation supports antitumor immunity, while preserving muscle mass, and found that HMB has muscle-sparing and antitumor activities against PDAC in obese mice. Moreover, our preclinical studies indicated that HMB sensitizes PDAC to immunotherapy.

## 2. Materials and Methods

### 2.1. Mice and Dietary Interventions

All experiments were approved by the Institutional Animal Care and Use Committee at the University of Texas at Austin or the University of North Carolina at Chapel Hill. Study 1 determined the effect of diet-induced obesity (DIO) and HMB supplementation on PDAC tumor growth. Study 2 determined the effect of HMB supplementation on anti-programed death 1 (PD1) immunotherapy response. Male C57BL/6 mice were obtained from Charles River Breeding Laboratories at 6 to 8 weeks of age. Mice were placed on the control diet and allowed to acclimate for 1 week. For Study 1, mice were randomized to either continue on the control diet with 10% kcal from fat (D12450J from Research Diets) or receive the DIO diet with 60% kcal from fat (D12491 from Research Diets) for 10 weeks ad libitum. For Study 2, all mice were provided with the AIN-93G (D10012G) diet ad libitum.

All diets were purchased from Research Diets, Inc (New Brunswick, NJ, USA). Ca-HMB (HMB) was incorporated at 1% (w/w) of the diet to achieve a daily fed amount of ~0.5 g HMB/kg bodyweight (approximating a 6 g/day human dose [27]). For Study 1, dietary HMB supplementation began 2 days prior to tumor cell injection. Specifically, mice (*n* = 30/group) were randomized to receive a control diet (D12450J), control + HMB, DIO (D12491), or DIO + HMB. For Study 2, which also used 30 mice/diet group, HMB supplementation began 1 week prior to tumor cell injection, with mice randomized to receive either the control diet (which was AIN-93G for this study) or control + HMB.

### 2.2. Fasting Blood Glucose and Serum Collection

At week 10 on the diet for Study 1, all mice (*n* = 30/diet group) were fasted for 6 h and then blood was collected via tail nicks to assess fasting blood glucose levels as measured with a Contour glucometer (Bayer HealthCare LLC, Whippany, NJ, USA). This was repeated immediately prior to euthanasia. Blood was also collected prior to tumor injections via retro-orbital bleeds and incubated at room temperature for 30 min to coagulate, and were then centrifuged at 11,000 RCF for 5 min. Serum was separated, snap-frozen, and stored at −80 °C until used to measure circulating hormone levels.

### 2.3. Subcutaneous Panc02 Tumor Cell Injections

At the point of diet switch, all mice were subcutaneously injected into the right flank with 250,000 Panc02 murine PDAC cells in PBS (generously provided by Dr. J. Schlom, National Cancer Institute, Bethesda, MD, USA). Beginning 1 week after tumor cell injections, tumors were measured biweekly with calipers.

### 2.4. Gemcitabine Treatment

In Study 1, once the tumors were palpable, all mice received either gemcitabine (50 mg/kg) or vehicle injections intraperitoneally every 3 days. At week 15, all mice were fasted for 6–8 h and then euthanized by CO_2_ inhalation followed by cervical dislocation.

### 2.5. Anti-PD1 Treatment

In Study 2, once the tumors were palpable, all mice received either anti-PD1 or isotype control (200 µg/mouse) injections intraperitoneally every 3 days. After 3 weeks, all mice were then euthanized by CO_2_ inhalation followed by cervical dislocation.

### 2.6. Tissue Harvest

Once tumors reached 1.5 cm in any direction in any group, all remaining mice were euthanized. Tumors and gastrocnemius muscles were harvested and either snap-frozen in liquid nitrogen and stored at −80 °C, or fixed with 10% neutral-buffered formalin for 48 h before paraffin embedding.

### 2.7. Cell Culture

Panc02 cells were cultured in DMEM supplemented with 10% FBS and 2 mM glutamine (Thermo Scientific, Waltham, MA, USA). All cell lines were negative when tested for mycoplasma using the Universal Mycoplasma Detection Kit (ATCC, Manassas, VA, USA).

C2C12 cells (generously provided by Dr. E. Mills at the University of Texas at Austin) were maintained in culture at <70% confluence in DMEM supplemented with 20% FBS and 2 mM glutamine. For the differentiation experiments, C2C12 cells were seeded at 90% confluence. After 18 h, the medium was removed and replaced with DMEM supplemented with 2% horse serum (Sigma, St. Louis, MO, USA) and 2 mM glutamine. The medium was replaced every second day for 6 days and then C2C12 cells were examined morphologically for myotube formation. For the 18 h serum starvation experiments, C2C12 cells were cultured using DMEM supplemented only with 2 mM glutamine and treated with or without HMB (4 mM) (Sigma, St. Louis, MO, USA) and TNFα (10 ng/mL) (Peprotech, Cranbury, NJ, USA).

Bone marrow-derived macrophages (BMDM) were differentiated as previously described [28]. To induce M1-like polarization, BMDM were treated with 10 ng/mL LPS for 48 h in the presence or absence of HMB (1 mM).

### 2.8. Serum Hormones

Circulating hormone levels were analyzed using serum collected in week 10 (before dietary HMB supplementation and tumor cell injection). Insulin levels were measured using the Bio-Plex Pro Mouse Diabetes Insulin Single Plex Assay (Bio-Rad, Hercules, CA, USA) and insulin-like growth factor (IGF)-1 levels were measured using the Millipore MILLIPLEX Rat/Mouse IGF-1 Single Plex Assay (Millipore, Burlington, MA, USA). Analysis was performed using the Bio-Plex 100 Analysis System (Bio-Rad, Hercules, CA, USA).

### 2.9. Tumor and Muscle Hematoxylin and Eosin Staining and Immunohistochemistry

Paraffin-embedded tumors and gastrocnemius muscles were cut into 4-µm-thick sections and processed for either hematoxylin and eosin (H&E) or immunohistochemical (IHC) staining. The gastrocnemius muscle tissue was cut cross-sectionally to quantify the thickness of each individual fiber. Staining was performed at the Histology Core Laboratory at the University of Texas-MD Anderson Cancer Center, Science Park Research Division (Smithville, TX, USA).

For IHC, antigen retrieval was achieved by microwaving slides with a 10 mM citrate buffer. Nonspecific binding was blocked by treating sections with Biocare blocking reagent (Biocare Medical, Pacheco, CA, USA) for 30 min at room temperature, followed by incubation with the primary antibody diluted in a blocking buffer overnight at 4 °C. The following primary antibodies and dilutions were used: phospho-S6 ribosomal protein S235/236 (Cell Signaling, 1:100) and Ki-67 (Dako, 1:100).

Images were captured by the Aperio ScanScope XT (Aperio Technologies, Vista, CA, USA) and staining was quantified using the Aperio ImageScope (Aperio Technologies, Vista, CA, USA). Automated algorithms were used to determine positive nuclear staining of Ki-67 and positive cytoplasmic staining of phospho-S6. The percentage of positive cells (or positive intensity) was obtained with a 10× objective in four different areas of the PDAC sections. Necrotic sections were excluded. Positive staining was averaged per treatment group.

### 2.10. Gene Expression Microarray Analysis

Total RNA was extracted using TRIzol and the RNeasy Kit (Qiagen, Hilden, Germany) from homogenized tumor samples, and RNA quality was assessed using an Agilent bioanalyzer (Agilent Technologies, Santa Clara, CA, USA). The Affymetrix Mouse Gene 2.1 ST 24-Array plate was used for Study 1, and the Clariom S array was used for all other samples. Feature selection for PCA and hierarchical clustering was conducted using F or *t* statistics.

Gene Set Enrichment Analysis (GSEA) using the Hallmark and GO biological process curated gene sets in the Molecular Signatures Database (MSigDB) was run with the default criteria and 1000 permutations [29,30].

Digital cytometry was performed using CIBERSORTx [31], using the mouse reference signatures obtained from seq-immuCC [32] and 1000 permutations.

### 2.11. Mass Cytometry

Tumors were dissociated using the Miltenyi mouse tumor dissociation kit, and CD45+ cells were enriched using Percoll differential centrifugation. Live cells were stained using Cell-ID cisplatin, and Fc receptors were blocked with TruStain FcX (1:100) prior to extracellular staining using the University of North Carolina mass cytometry core antibody panel. Cells were fixed and permeabilized for intracellular staining of FoxP3 using the eBioscience FoxP3 (ThermoFisher Scientific, Waltham, MA, US) staining buffer set. Finally, cells were intercalated with iridium overnight. Live CD45+ single cells were gated using Cytobank software (version 9.0, Beckman Coulter, Brea, CA, USA) and then analyzed in R as previously described [33]. Briefly, following hyperbolic inverse sine transformation, a self-organizing map was built, and consensus clustering was performed. Over-clustered clusters were collapsed manually and visualized by tSNE plots. The differential abundance of each cell type and the differential expression of each marker with each cell type was determined by a generalized linear mixed logistic model.

### 2.12. Quantitative RT-PCR

RNA was isolated from differentiated C2C12 myotubes using the E.Z.N.A. total RNA extraction kit (Omega Bio-Tek, Norcross, GA, USA). Total RNA was reverse-transcribed to cDNA using the High-Capacity cDNA Reverse Transcription Kit (ThermoFisher Scientific, Waltham, MA, US). Gene expression was normalized to the housekeeping gene ß-actin. Relative differences in gene expression were analyzed using the 2^-ΔΔCT^ method. All primers used are listed in Table S1.

### 2.13. Muscle Fiber Size

Cross-sectional images of H&E-stained gastrocnemius muscle were analyzed using Fiji (ImageJ Version 1.53n from the NIH). Prior to analysis, the Advanced Weka Segmentation classifier included in Fiji was trained by manually marking regions of the small and dark slow-twitch fibers, the large and light fast-twitch fibers, and the slide background [34]. Muscle fiber size was determined using the minimal Feret’s diameter [35]. Randomly selected slides (*n* = 6 mice/group), with 100–400 randomly selected muscle fibers per slide, were analyzed. The minimal Feret’s diameters of each fiber were averaged for each treatment group.

### 2.14. Western Blots

Cells were homogenized in a radioimmunoprecipitation (RIPA) buffer with protease and phosphatase inhibitors and incubated on ice for 30 min. Samples were then centrifuged at 16,000 RCF for 15 min at 4 °C, and the supernatant was transferred to a new tube.

Protein lysates were resolved using a 4–16% gradient of polyacrylamide gels and transferred to nitrocellulose membranes (Sigma, St. Louis, MO, USA). The following primary antibodies and dilutions were used: myosin heavy chain (1:1000; R&D Systems, Minneapolis, MN, USA), phospho-p65^S536^ (1:1000; Cell Signaling Technology, Danvers, MA, USA), p65 (1:1000; Cell Signaling Technology, Danvers, MA, USA), ß-actin (1:10,000; Santa Cruz, Dallas, TX, USA), α-tubulin (1:10,000; Santa Cruz, Dallas, TX, USA), and histone H3 (1:1000; Cell Signaling Technology, Danvers, MA, USA). Membranes were incubated for 1 h at room temperature in species-specific secondary antibodies (LI-COR) diluted 1:5000 in 5% BSA in TBS-T.

Membranes were scanned and densitometry was assessed using the Odyssey infrared fluorescent imaging system using LI-COR software (version 5.2, LI-COR, Lincoln, NE, USA). Raw values were compared between groups only if samples were on the same membrane. Raw values normalized to loading control levels were used to calculate the relative protein levels.

### 2.15. Nuclear and Cytoplasmic Fractionation of C2C12 Myotubes

To generate nuclear and cytoplasm-enriched fractions, 5 × 10^6^ C2C12 myotubes were incubated in a hypotonic fractionation buffer containing 10 mM HEPES (pH 7.4), 10 mM KCl, 1 mM MgCl2, 1 mM EDTA, 1 mM EDGA, 1 mM DTT, and protease and phosphatase inhibitors on ice for 20 min. Cells were then sheared by passing the solution 5 times through a 17-gauge needle and then incubating them on ice for an additional 10 min. The cytoplasmic fraction was obtained by centrifuging the solution at 710 RCF for 5 min and retaining the supernatant. The nuclear fraction was then washed with a hypotonic fractionation buffer, cycling it through a 15-gauge needle 10 times. The nuclear fraction was centrifuged at 20,000 RCF for 5 min and the pellet was lysed in a RIPA lysis buffer and sonicated. Histone H3 and α-tubulin were used as purity controls for the nuclear and cytoplasmic fractions, respectively.

### 2.16. Statistical Analyses

Statistical analyses were conducted using GraphPad Prism (GraphPad Software 9.0.0, San Diego, CA, USA). Comparisons between the two groups were analyzed using unpaired, two-tailed t-tests. Comparisons among more than two groups were analyzed using one-way ANOVA. Associations between HMB treatment and muscle fiber size and CD3 staining were determined by two-way ANOVA. Principal components analysis (PCA) and hierarchical clustering was performed using R (version 4.0.2, R Core Team, Vienna, Austria).

## 3. Results

### 3.1. DIO Increases Bodyweight and Circulating Glucose, Insulin, and IGF-1 Levels

To determine whether HMB alters obesity-exacerbated PDAC tumor growth, we fed male C57BL/6 mice the control diet (10% kcal fat) or a high-fat DIO diet (60% kcal fat) to promote obesity. Following 10 weeks of the diet treatment, we confirmed that mice had achieved the metabolic characteristics expected of DIO. Relative to the control mice, DIO mice had significantly greater bodyweight, fasting blood glucose, and serum levels of insulin and IGF-1 (Figure S1A–D). Subsequently, mice were randomized to either continue on their respective diets or receive the same diets with HMB supplementation (control + HMB and DIO + HMB), and also received PDAC tumor injections. HMB supplementation did not alter terminal bodyweight in either diet group (Figure S1E).

### 3.2. HMB Supplementation Reduces Transplanted PDAC Growth in DIO Mice Independent of Gemcitabine Treatment, Glucose Levels, and mTOR Activation

**Figure 1:**
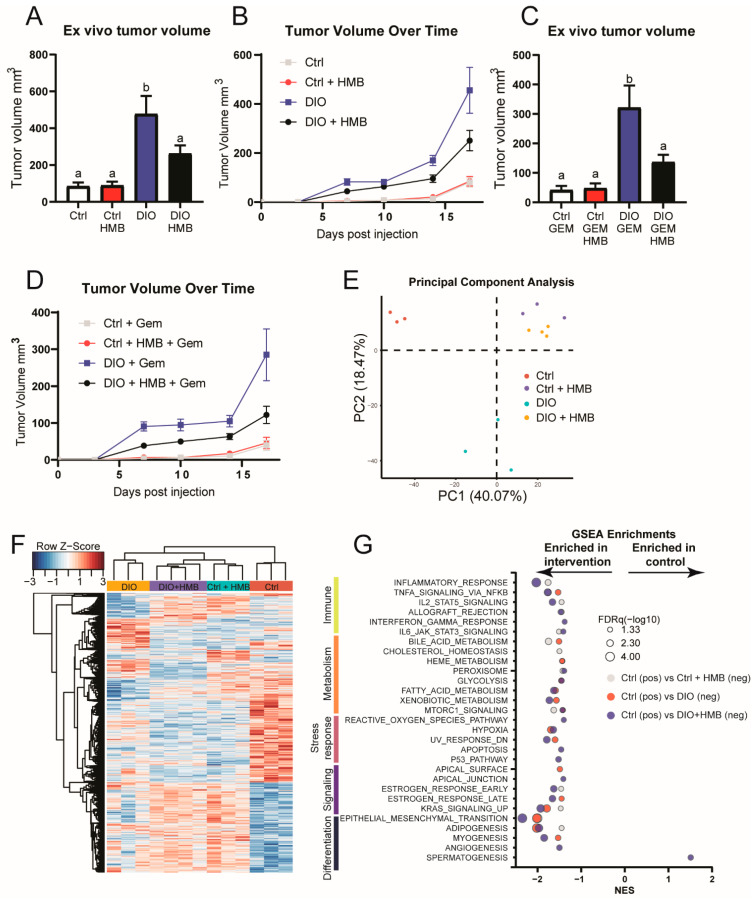
(**A**,**B**) Tumor volume in control and DIO mice with or without HMB supplementation at study termination (**A**) or over time (**B**) (*n* = 14/group). (**C**,**D**) Gemcitabine-treated tumor volume in control and DIO mice with or without HMB supplementation at study termination (**C**) or over time (**D**) (*n* = 13–15/group). (**E**–**G**) Transcriptomic analysis of tumors from control and DIO mice with or without HMB supplementation. (**E**) Principal component analysis (*n* = 3–4/group) and (**F**) hierarchical clustering (*n* = 3–4/group). (**G**) Significant GSEA enrichments from Hallmark gene sets resulting from comparing control vs. control + HMB (gray), control vs. DIO (red), and control vs. DIO + HMB (blue) mice. FDRq is denoted by the bubble size. All data are presented as means ± SEM. Differences are considered significant if *p* < 0.05, as indicated by different letters within the same graph.

DIO mice had an increased tumor volume relative to the control mice (Figure 1A,B). DIO + HMB mice had a reduced tumor volume relative to DIO mice; however, the tumor volume in control + HMB mice was not altered relative to that in control mice.

When treated with gemcitabine (50 mg/kg every third day), a common PDAC chemotherapeutic agent, DIO mice continued to have an elevated tumor volume compared with control mice. HMB supplementation significantly reduced tumor size in DIO mice such that tumor mass was not different than that of the unsupplemented control (Figure 1C,D). HMB supplementation did not alter blood glucose in either lean or obese mice (Figure S2A). Tumoral Ki-67 expression, a marker of proliferation, was increased in tumors from DIO mice relative to tumors from control mice, while tumors from the DIO + HMB mice displayed Ki-67 expression levels intermediate to the control and DIO groups (Figure S2B). However, gemcitabine treatment did not affect Ki-67 expression (Figure S2C). Given that HMB indirectly promotes mTOR activation via a number of mechanisms [36], we assessed tumor mTOR activity. HMB did not alter mTOR activity, as reflected by the extent of S6 phosphorylation in control and DIO mice, irrespective of gemcitabine treatment (Figure S2D,E).

### 3.3. HMB Partially Reverses DIO-Associated Changes in Gene Expression in the Tumor Microenvironment

We performed microarray transcriptomic analysis on tumors from control and DIO mice with and without HMB supplementation. Principal component analysis and hierarchical clustering of our transcriptomic data, following feature selection using differential gene expression analysis, revealed a clear separation in gene expression in tumors from control and DIO mice, as well as separation between tumors from the HMB-treated and untreated groups (Figure 1E,F).

We next utilized GSEA to stratify differential gene expression by physiological processes, using the curated Hallmark gene sets [30]. Normalized enrichment scores and the associated FDRq values for the binary comparisons (control vs. control + HMB, control vs. DIO, control vs. DIO + HMB, and DIO vs. DIO + HMB) are listed in Table S2. The GSEA analysis comparing tumors from control and DIO mice revealed that DIO modulated numerous cancer-associated pathways, including hypoxia, KRAS signaling, and the epithelial-to-mesenchymal transition (EMT), as well as one immune-related gene set. Control + HMB mice, relative to the unsupplemented control, increased three immune-related gene sets as well as several other gene sets, including cholesterol metabolism and KRAS signaling. The comparison of control and DIO + HMB mice resembled the comparison of the control with either factor in isolation, with enrichment of six out of a total of seven available immune-related gene sets (Figure 1G). The tumoral sensitivity of immune-related pathways to HMB treatment was confirmed in the comparison of DIO vs. DIO + HMB mice, in which allograft rejection was the only gene set enriched in tumors from HMB treated mice (Table S2).

**Figure 2:**
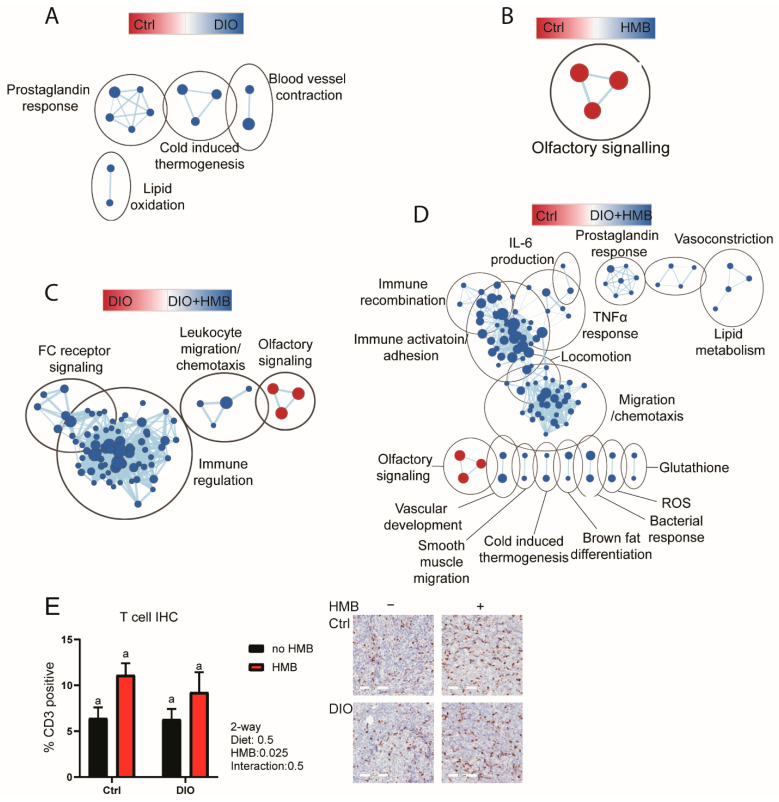
(**A**–**D**) Enrichment maps of significant GSEA GO biological processes in tumors from control and DIO mice supplemented with and without HMB. (FDRq < 0.05). Node color indicates the normalized enrichment score, node size indicates the gene set size, line weight indicates the degree of overlap, and clusters indicate the minimum 50% overlap of gene sets. (**A**) Control vs. DIO, (**B**) control vs. control + HMB, (**C**) DIO vs. DIO + HMB, (**D**) control vs. DIO + HMB (*n* = 3–4/group). (**E**) T cell quantification by immunohistochemistry staining of CD3 (*n* = 5–6/group). Scale bars represent 100 µm.

We next analyzed our transcriptomic data using GO biological process gene sets using the same binary comparisons. Several hundred gene sets were differentially enriched (Table S2). To reduce redundancy between these gene sets [37], we mapped the resulting data as enrichment maps and clustered them by gene set overlap. In agreement with our initial Hallmark gene sets, tumors from DIO mice were enriched for gene sets relating to prostaglandin signaling and metabolism relative to the control (Figure 2A). Suppression of olfactory signaling by HMB was the only enriched cluster detected when contrasting control vs. control + HMB tumors (Figure 2B). The overwhelming majority of gene sets and clusters enriched in DIO + HMB tumors, when compared with DIO tumors, were immune-related, and were all enriched in the HMB-supplemented group (Figure 2C). The comparison of control vs. DIO + HMB mice indicated that >100 gene sets, many immune- or metabolism-related (Table S2), were enriched in DIO + HMB mice (Figure 2D). We also stained tumor sections for CD3 to determine tumor CD3+ T cell abundance using CD3 IHC. While CD3+ T cell abundance was not different among groups, a two-way ANOVA indicated a significant positive association between HMB and CD3+ T cell numbers (Figure 2E).

### 3.4. HMB Supplementation Promotes a Response to Anti-PD1 Immune Checkpoint Inhibition

**Figure 3:**
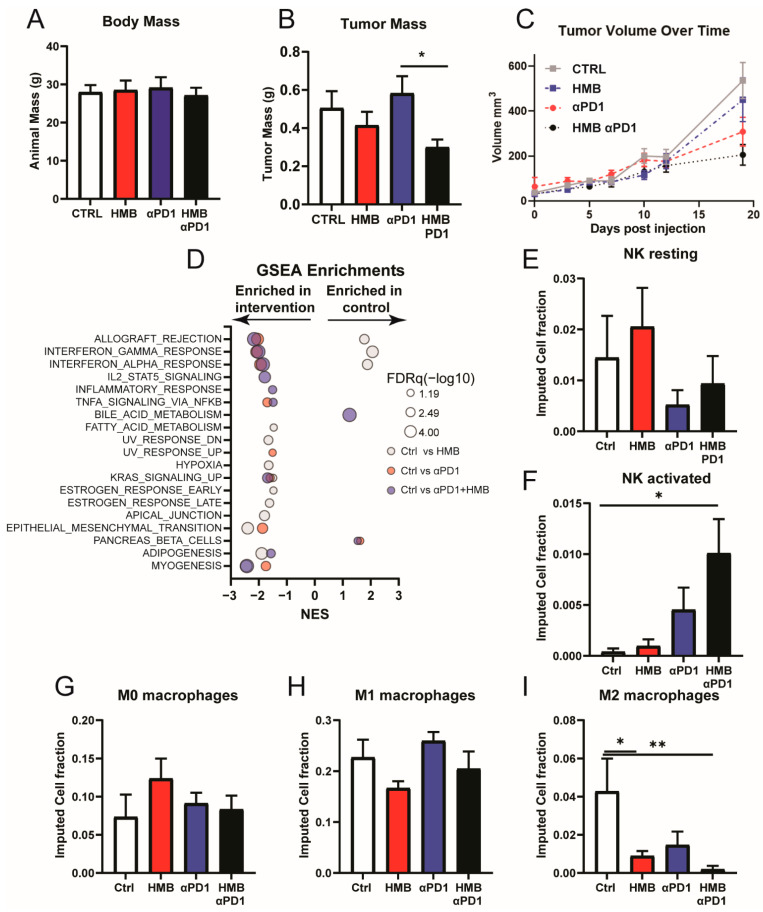
(**A**) Body mass of mice supplemented with or without HMB and treated with or without anti-PD1 immunotherapy at study termination (*n* = 12–13/group). (**B**) Tumor mass in mice supplemented with or without HMB and treated with or without anti-PD1 immunotherapy at study termination (*n* = 12–13/group). (**C**) Tumor volume over time of mice supplemented with or without HMB and treated with or without anti-PD1 immunotherapy (*n* = 12–13/group). (**D**–**I**) Transcriptomic analysis of tumors from control and anti-PD1-treated mice with or without HMB supplementation (*n* = 6–7/group). (**C**) Significant GSEA enrichments from Hallmark gene sets resulting from comparing control vs. HMB (gray), control vs. PD1 (red), and control vs. PD1 + HMB (blue) mice. FDRq is denoted by the bubble size. (**D**–**H**) CIBERSORTx digital cytometry-imputed cell fractions: (**D**) resting NK cells, (**E**) activated NK cells, (**F**) M0 macrophages, (**G**) M1-like macrophages, and (**H**) M2-like macrophages. All data are presented as means ± SEM. Differences are considered significant if *p* < 0.05 (* *p* < 0.05, ** *p* < 0.01).

Since PDAC has thus far proven unresponsive to available immunotherapies [1,38], we hypothesized that HMB would promote a response in these tumors, similar to its effects in DIO mice. Hence, we subcutaneously injected Panc02 cells into mice fed a control diet (AIN-93G) with (control + HMB) or without (control) 1% HMB supplementation. Two weeks following tumor injection, we treated these mice with or without anti-PD1 immunotherapy (200 µg/mouse every third day). We assessed the toxicity of anti-PD1 or the combination of anti-PD1 + HMB via bodyweight measurements but did not observe any differences among groups (Figure 3A). Neither HMB nor anti-PD1 alone altered tumor mass. However, the combination of anti-PD1 + HMB resulted in a reduced tumor burden relative to the anti-PD1-only group (Figure 3B,C).

We next used GSEA to determine pathway activation within the tumors’ transcriptomic data. All enrichment scores and associated FDRq values are listed in Table S3.

The comparison of anti-PD1 vs. control indicated modest activation of immune-related gene sets (four of seven), along with limited induction of other cellular metabolic or signaling responses (Figure 3D). The comparison of control vs. HMB indicated once again that HMB promoted the activation of various cellular signaling and metabolic pathways, including estrogen signaling and fatty acid metabolism. In contrast to our previous finding of the modest activation of immune-related gene sets, we observed the suppression of three of seven immune-related gene sets following HMB treatment. However, the comparison of control vs. anti-PD1 + HMB indicated robust activation of anti-tumor immunity (six of seven immune-related gene sets), with limited activation of other pathways. Digital cytometry using CIBERSORTx revealed no difference in the resting NK cell population but an increased number of activated NK cells in anti-PD1 + HMB compared with the control (Figure 3E,F). Further, M0-like and M1-like macrophage populations were not altered; however, M2-like macrophage abundance was reduced in both HMB and anti-PD1 + HMB compared with the control (Figure 3G–I).

**Figure 4:**
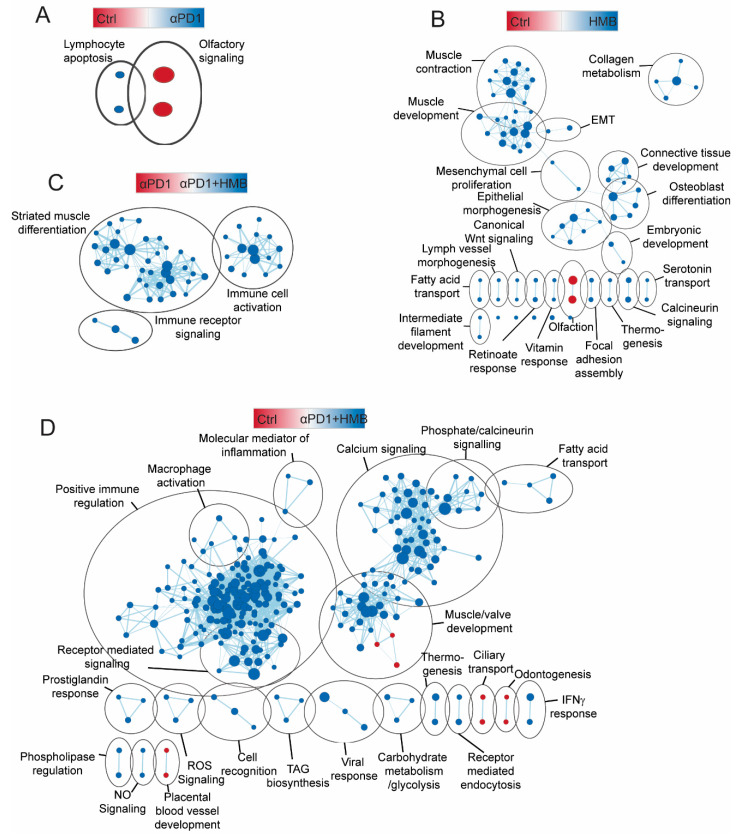
(**A**–**D**) Enrichment map of significant GSEA GO biological processes in tumors from Control and anti-PD1 treated mice supplemented with and without HMB (FDRq < 0.05). Node color indicates normalized enrichment score, node size indicates gene set size, line weight indicates degree of overlap, and clusters indicate minimum 50% overlap of gene sets. (**A**) Control vs αPD1, (**B**) Control vs HMB, (**C**) αPD1 vs αPD1 + HMB, (**D**) Control vs αPD1 + HMB (*n* = 6–7/group).

Once again, to gain a more granular insight into pathways activated by HMB, anti-PD1, or the combination of both treatments, we applied GSEA using the GO biological process gene sets, followed by enrichment mapping. All enrichment scores and the associated FDRq values are listed in Table S3. This approach confirmed that anti-PD1 alone compared with the control promoted limited pathway alteration (Figure 4A). HMB supplementation alone, compared with the control, promoted the activation of numerous gene sets and gene set clusters regulating differentiation, signaling, and metabolism; however, we did not observe the activation of any immune-related gene set clusters (Figure 4B). A comparison of anti-PD1 vs. anti-PD1 + HMB confirmed that HMB promotes the activation of immune- and muscle-related gene sets (Figure 4C). The combination of anti-PD1 + HMB, compared with the control, promoted the activation of ~180 gene sets, which clustered into numerous immune- and metabolic-related pathways (Figure 4D).

### 3.5. HMB Supplementation Promotes M1-Like Macrophage Activation

**Figure 5:**
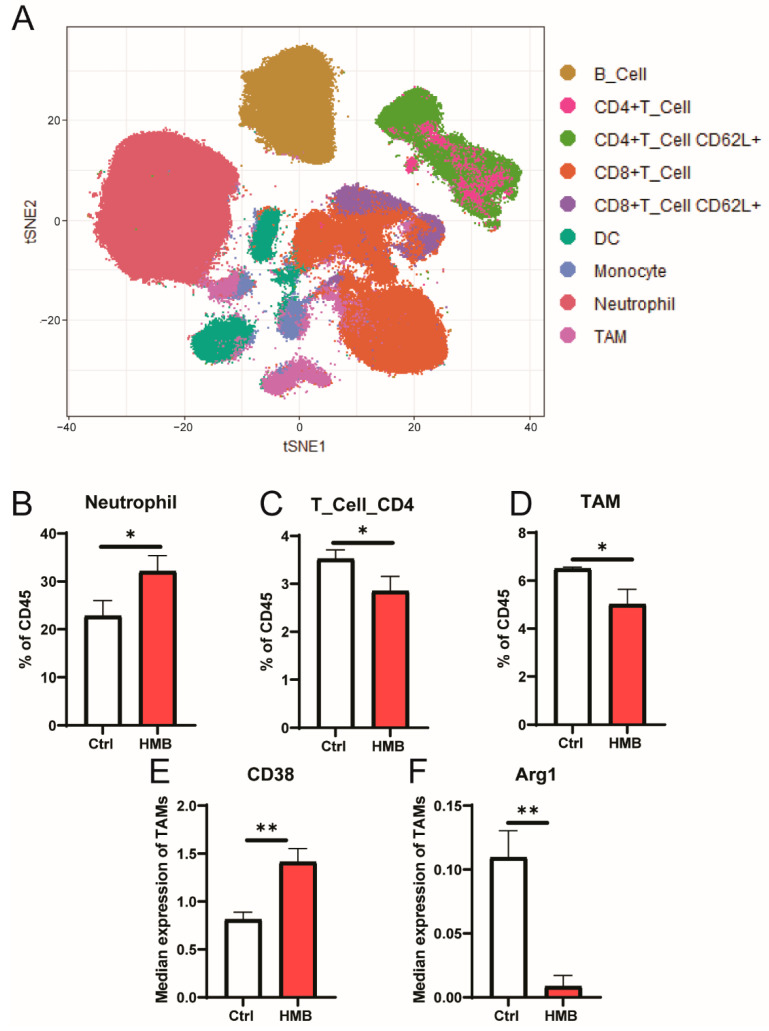
(**A**) tSNE visualization of tumor immune cells following clustering via FlowSOM. (**B**–**D**) Differential abundance of neutrophils, CD4+ T cells, and tumor-associated macrophages (TAMs) in tumors from control or HMB-supplemented mice. (**E**,**F**) Differential expression of CD38 and Arg1 in TAMs from tumors from control or HMB-supplemented mice (*n* = 3–4/group). Differences between groups considered significant if *p* < 0.05 (* *p* < 0.05, ** *p* < 0.01).

To determine whether HMB supplementation alters the tumor immune profile, we performed mass cytometry on tumors from control and HMB-supplemented mice. The distribution of cell populations was visualized using tSNE plots following clustering by FlowSOM (Figure 5A). Of the nine identified cell populations, three were differentially abundant between the control and HMB supplementation groups: neutrophils (Figure 5B), CD4+ T cells (Figure 5C), and tumor-associated macrophages (Figure 5D). We next assessed whether HMB supplementation altered TAM polarization in vivo. TAM expression of the M1-like marker CD38 was increased by HMB supplementation (Figure 5E), and expression of the M2-like marker Arg1 was reduced by HMB supplementation (Figure 5F).

**Figure 6:**
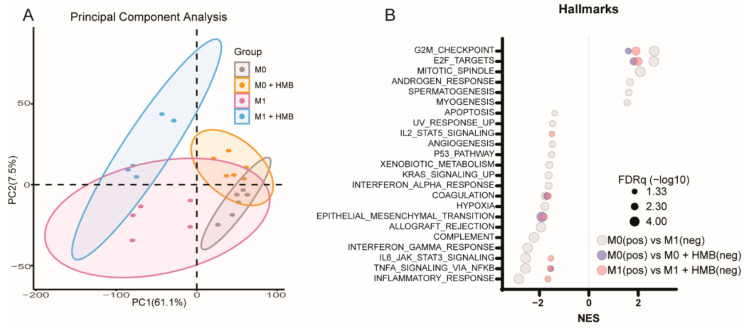
Transcriptomic analysis of bone marrow-derived M0- and M1-like macrophages supplemented with or without HMB (**A**,**B**). (**B**) Principal component analysis of M0/M1 macrophages treated with HMB (*n* = 5–6/group). Significant (FDRq < 0.05) GSEA enrichments from Hallmark gene sets resulting from comparing M0 vs. M1 (gray), M0 vs. M0 + HMB (red), and M1 vs. M1 + HMB (blue); FDRq is denoted by the bubble size.

We sought to directly test whether HMB promotes putatively antitumor M1-like macrophage polarization in vitro. M1-like macrophages were differentiated from bone marrow-derived macrophages using 10 ng/mL LPS for 48 h in the presence or absence of HMB. M0 bone marrow-derived macrophages were treated with HMB for 48 h. Principal component analysis of the transcriptomic data revealed partial separation between non-HMB-treated versus HMB-treated M0 and M1 macrophages (Figure 6A). GSEA analysis was conducted using the Hallmark gene sets to identify the pathways induced by HMB treatment in either M0- or M1-like macrophages. GSEA enrichments in the comparison between M0- and M1-like macrophages were used to identify M1-like pathway activation. All normalized enrichment scores and the associated FDRq values are presented in Table S4. HMB treatment in both M0- and M1-like macrophages promoted similar pathway enrichments which overlapped considerably with M0- to M1-like polarization (Figure 6B). HMB treatment promoted pathway activation which entirely overlapped with M1-like pathway activation, except for the epithelial-to-mesenchymal transition in M0 macrophages, and the epithelial-to-mesenchymal transition and angiogenesis in M1 macrophages, all of which were reduced by HMB supplementation. Thus, we concluded that HMB promotes M1-like polarization in M0 macrophages and enhances it in M1-like macrophages following LPS stimulation.

### 3.6. HMB Suppresses Muscle Inflammatory Signaling In Vivo and In Vitro to Enhance Myogenesis

**Figure 7:**
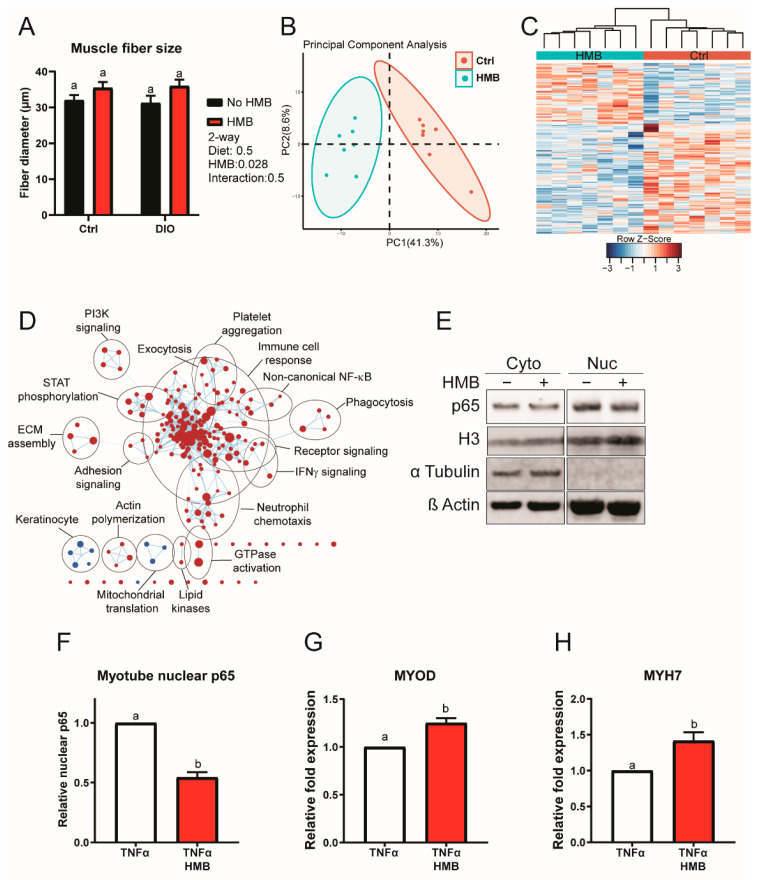
(**A**) Minimal Feret’s diameter of the gastrocnemius muscle measured on H&E sections from control and DIO mice with or without HMB supplementation (*n* = 6/group). (**B**–**D**) Transcriptomic analysis of the gastrocnemius muscle from control mice with or without HMB supplementation. (**B**) Principal component analysis (*n* = 7/group) and (**C**) hierarchical clustering (*n* = 7/group). (**D**) Enrichment map of significant GSEA GO bioprocesses enrichments (FDRq < 0.05). Node color indicates the normalized enrichment score (Control = red, HMB = blue), node size indicates the gene set size, line weight indicates the degree of overlap, and clusters indicate the minimum 50% overlap of gene sets. (**E**–**H**) C2C12 myotubes treated with HMB for 18 h in serum-free media containing 10 ng/mL TNFα. (**E**) Western blot of cytosolic and nuclear levels of NF-κB p65 (*n* = 4/group). (**F**) Quantification of nuclear NF-κB p65 protein levels (*n* = 4/group). mRNA expression of myogenesis markers (**G**) *Myod* and (**H**) *Myh7* following normalization to ß-actin (*n* = 4/group). All data are presented as means ± SEM. Differences are considered significant if *p* < 0.05, as indicated by different letters within the same graph. The uncropped Western blots have been shown in Figure S4.

Given the important associations between muscle mass and antitumor immunity, and HMB’s muscle-protective effects, we sought to determine if our models reflected the muscle-protective effects of HMB. While HMB did not alter muscle fiber size in either the control or DIO groups in Study 1, HMB supplementation was associated with increased gastrocnemius muscle size across both diet groups (*p* = 0.029) (Figure 7A). Given this finding, we sought to determine whether HMB modulated inflammatory signaling in muscle in another cohort of mice. We tested this using transcriptional profiling of RNA isolated from gastrocnemius muscle of control and control + HMB mice in Study 2. The control and control + HMB groups clustered separately via PCA and hierarchical clustering (Figure 7B,C).

Analysis of pathway activation by GSEA using the Hallmark gene sets revealed that HMB supplementation suppressed proinflammatory signaling while promoting oxidative phosphorylation (Figure S3). To further interrogate these data, we the GSEA GO bioprocess gene sets and visualized the results using enrichment mapping. In accordance with the Hallmark gene set analysis, the GO bioprocess gene sets also revealed that HMB was associated with the suppression of numerous inflammatory processes, as well as promotion of mitochondrial translation (Figure 7D; Table S5).

Having established that HMB protects muscle mass and suppresses inflammation in vivo (including TNFα signaling), we sought to determine whether HMB could directly antagonize pro-inflammatory signaling through NF-κB in C2C12 myotubes in vitro. To test this, we differentiated C2C12 murine muscle cells, as confirmed by myosin heavy chain expression, and subsequently treated the myotubes with 10 ng/mL TNFα in serum-free media with or without 4 mM HMB for 18 h. To confirm a reduction of NF-κB activity we performed nuclear cytosolic fractionation, employing histone H3 and α-tubulin as purity controls for nuclear and cytoplasmic fractions, respectively. Nuclear localization of p65 following TNFα treatment was reduced, indicating significant inhibition of NF-κB activity (Figure 7E,F, Figure S4). Simultaneously, gene expression of Myogenic Differentiation 1 (*Myod*, a myogenic transcription factor suppressed by NF-κB [39]), and Myosin Heavy Chain 7 (*Myh7*, terminal muscle differentiation marker) was upregulated as determined by qPCR (Figure 7G,H).

## 4. Discussion

Our findings demonstrate that dietary HMB supplementation decreases in vivo growth of PDAC in DIO mice, promotes antitumor immunity in DIO mice, and improves immunotherapy response in control mice. More specifically, we show that HMB supplementation not only remodels the pro-tumorigenic microenvironment associated with DIO to promote antitumor immunity but also synergizes with anti-PD1 immunotherapy and promotes M1-like polarization of macrophages. Our results also indicate that HMB antagonizes inflammatory signaling through NF-κB in myotubes in vitro and muscle in vivo. Taken together these data suggest that HMB supplementation may promote antitumor immunity while reducing inflammatory signaling in muscle. These findings are important given the current dearth of effective PDAC therapy in the clinic, the prevalence of muscle wasting in patients with PDAC, and the well-established anabolic utility of HMB supplementation [40].

The obesity epidemic, the rising prevalence of obesity-associated PDAC, and the increasing proportion of PDAC patients that are obese at the start of therapy lend considerable urgency to better understanding obesity and PDAC [8]. Obesity [14,15,16] and maintenance of muscle mass [6,41,42] both independently predict a favorable response to immune checkpoint inhibition therapy. Separately, sarcopenic obesity is linked with increased morbidity and mortality in patients with PDAC due at least in part to its association with cancer-associated cachexia [10]. Thus, obesity is a critical determinant of therapy response in PDAC. Here we have identified in our preclinical studies that HMB supplementation may be an effective, low-cost intervention that promotes antitumor immunity and preserves muscle mass in PDAC.

HMB supplementation exerted differential effects on muscle versus tumor. In the muscle, HMB supplementation promoted increased muscle fiber size and suppressed inflammatory signaling. Additionally, HMB antagonized NF-κB signaling and promoted myogenic gene expression in vitro. These data are consistent with a muscle protective role for HMB mediated in part via antagonizing inflammatory signaling. TNFα signaling through NF-kB is a pleotropic regulator of the immunotherapy response [23]. In muscle, NF-κB leads to muscle atrophy via increased protein degradation [25]. Inhibition of NF-κB prevents cancer-induced muscle atrophy [26]. NF-kB signaling is highly activated in an obese environment [43], which may explain why patients with sarcopenic obesity often have enhanced tumor growth coupled with muscle loss. Indeed, in a rat model of breast cancer, HMB decreased inflammatory signaling within cancer cells and protected against cachexia [20]. In our tumor-bearing control-fed mice, HMB had no effect on tumor size or tumor cell proliferation, and had limited effects on the tumor transcriptome. However, HMB significantly decreased the tumor size in DIO mice and DIO mice treated with gemcitabine. These HMB-mediated effects were independent of blood glucose levels or tumor mTOR activation. This contrasts with leucine, of which HMB is a metabolite, which promotes tumor growth and mTOR activation in PDAC [44].

To determine potential mediators of the antitumor effects of HMB, we analyzed the tumor transcriptome. GSEA revealed that the combination of HMB with the proinflammatory stimuli from either DIO or anti-PD1 promoted effective antitumor immunity, including increased T cells within tumors following HMB supplementation. HMB is also known to promote in vitro phagocytic function and respiratory burst formation in chicken macrophages [21], goat monocytes, and goat granulocytes [22]. In this study, we identified antitumor M1-like macrophage polarization in vivo and in vitro following HMB supplementation alone or in combination with anti-PD1. Immunosuppressive M2-like macrophages promote tumor growth via several mechanisms [45]. Thus, the finding that HMB supplementation promotes antitumor polarization of macrophages in vitro and in vivo suggests that HMB may promote the metabolic reprogramming of macrophages.

Although our PDAC transplant model provides insights into how HMB supplementation effects tumor growth, it cannot provide information with respect to tumor development. An additional limitation of our PDAC transplant model is that it does not induce cancer-associated cachexia. However, there are other well-established cachexia mouse models in which HMB has been demonstrated to prevent cancer-associated cachexia [46,47]. HMB has been shown to reduce cachexia, with a concomitant decrease in NF-κB, in a subcutaneous injection model of Walker-256 breast cancer cells in Sprague-Dawley rats [20]. Similarly, in an intraperitoneal injection model of Yoshida AH-130 ascites hepatoma cells in Wistar rats, HMB was again found to reduce cachexia, and this observation was associated with mTOR activation [47]. Thus, while our PDAC model does not induce cachexia, the muscle-protective effects we observed are consistent with the findings in other well-characterized cancer cachexia models.

## 5. Conclusions

This report established that: (1) HMB is associated with increased muscle fiber size and a reduction in muscle inflammation in control and DIO mice; (2) obesity significantly increases Panc02 tumor growth, which is antagonized by HMB; (3) gene expression profiling and immunohistochemistry indicate that HMB promotes antitumor immunity in control and DIO mice; (4) HMB synergizes with anti-PD1 immunotherapy in PDAC to elicit antitumor immunity; and (5) HMB promotes proinflammatory macrophage activation. These preclinical findings suggest that HMB has promising muscle-sparing, immune-enhancing, and antitumor activities against PDAC in the context of obesity and anti-PD1 immunotherapy. Given the well-established and minimal side-effect profile of HMB in cancer patient populations, this work positions HMB as a potential adjuvant to PDAC therapy.

## Data Availability

Transcriptomic data generated in this study are available at the Gene Expression Omnibus, accessions GSE120348, GSE178475, GSE178476, and GSE178477; the remaining data are available upon request from the corresponding author.

## Supplementary Materials

The following are available online at www.mdpi.com/article/10.3390/cancers13246359/s1. Figure S1: Metabolic effects of DIO and HMB supplementation, Figure S2: Blood glucose, proliferation, and apoptosis are not altered by HMB, Figure S3: HMB supplementation alters GSEA Hallmark enrichments in muscles, Figure S4: Uncropped Western blots for Figure 7E, Table S1: Primer sequences, Table S2: Study 1 GSEA enrichments, Table S3: Study 2 GSEA enrichments, Table S4: Bone marrow-derived macrophage GSEA enrichments, Table S5: Muscle GSEA enrichments.
