# Supplementary material for: β-Hydroxy-β-Methylbutyrate Supplementation Promotes Antitumor Immunity in an Obesity Responsive Mouse Model of Pancreatic Ductal Adenocarcinoma
Source: Cancers (Basel). 2021 Dec 18;13(24):6359. doi: 10.3390/cancers13246359 (PMC8699071; doi:10.3390/cancers13246359)
Supplement: Supplementary file 1 [file cancers-13-06359-s001.zip › cancers-1491645-Suppl Figures.pdf]

cancers

MDPI

Supplementary Materials: β-Hydroxy-β-Methylbutyrate Supplementation Promotes Antitumor Immunity in An Obesity Responsive Mouse Model of Pancreatic Ductal Adenocarcinoma

Michael F Coleman, Kristyn A Liu, Alexander J Pfeil, Suhas K Etigunta, Xiaohu Tang, Salvador Fabela, Laura M Lashinger, Zhengrong Cui and Stephen D Hursting

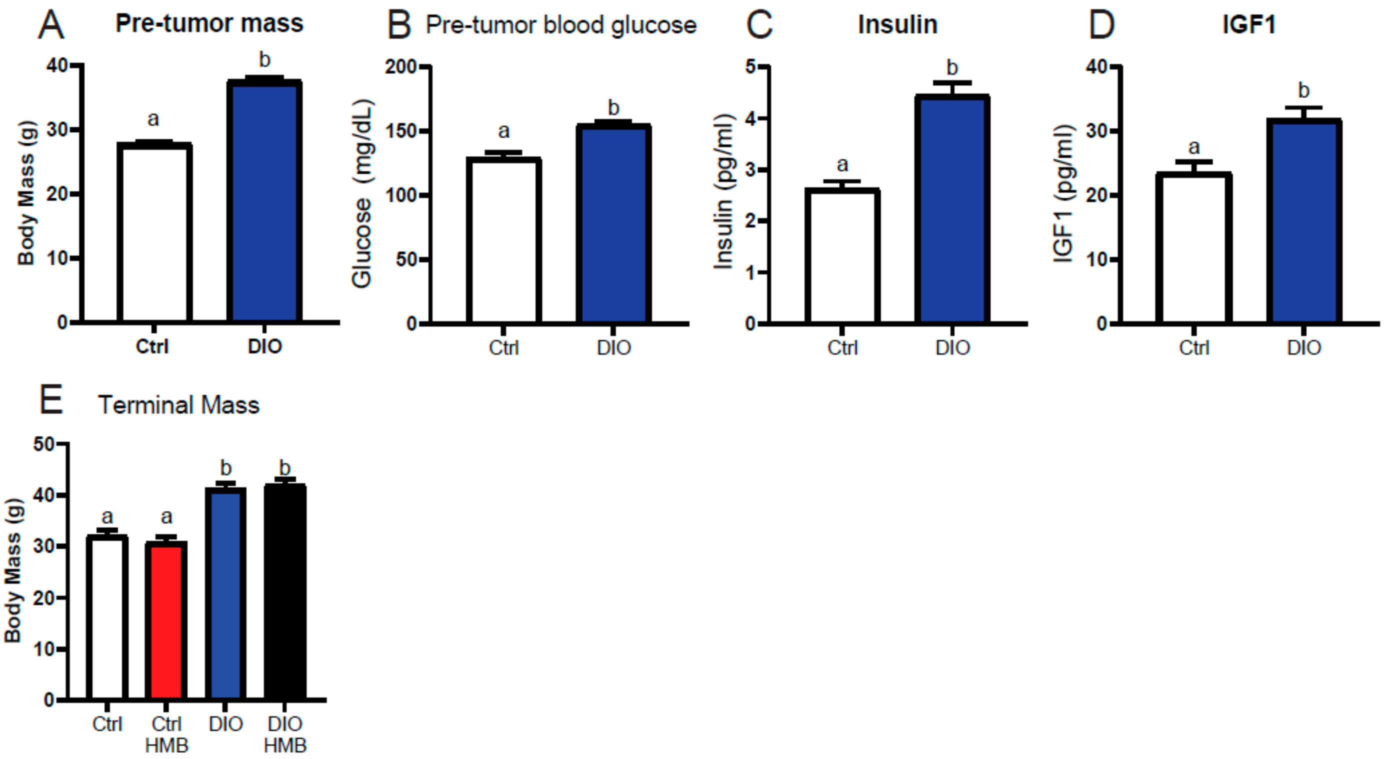

Figure S1. Metabolic effects of DIO and HMB supplementation. Following 10 weeks of DIO diet regimen metabolic alterations in control and DIO mice was assessed. (A) mouse body mass (n=28-30/group). (B-D) Blood glucose (n=28-30/group), serum insulin(n=26-30/group), and serum IGF-1(n=24-26/group) following 6 hour fast. (E) Terminal mouse body mass following HMB supplementation (n=14-15/group). All data are presented as mean ± SEM. Differences are considered significant if $p < 0.05$ as indicated by different letters within the same graph.

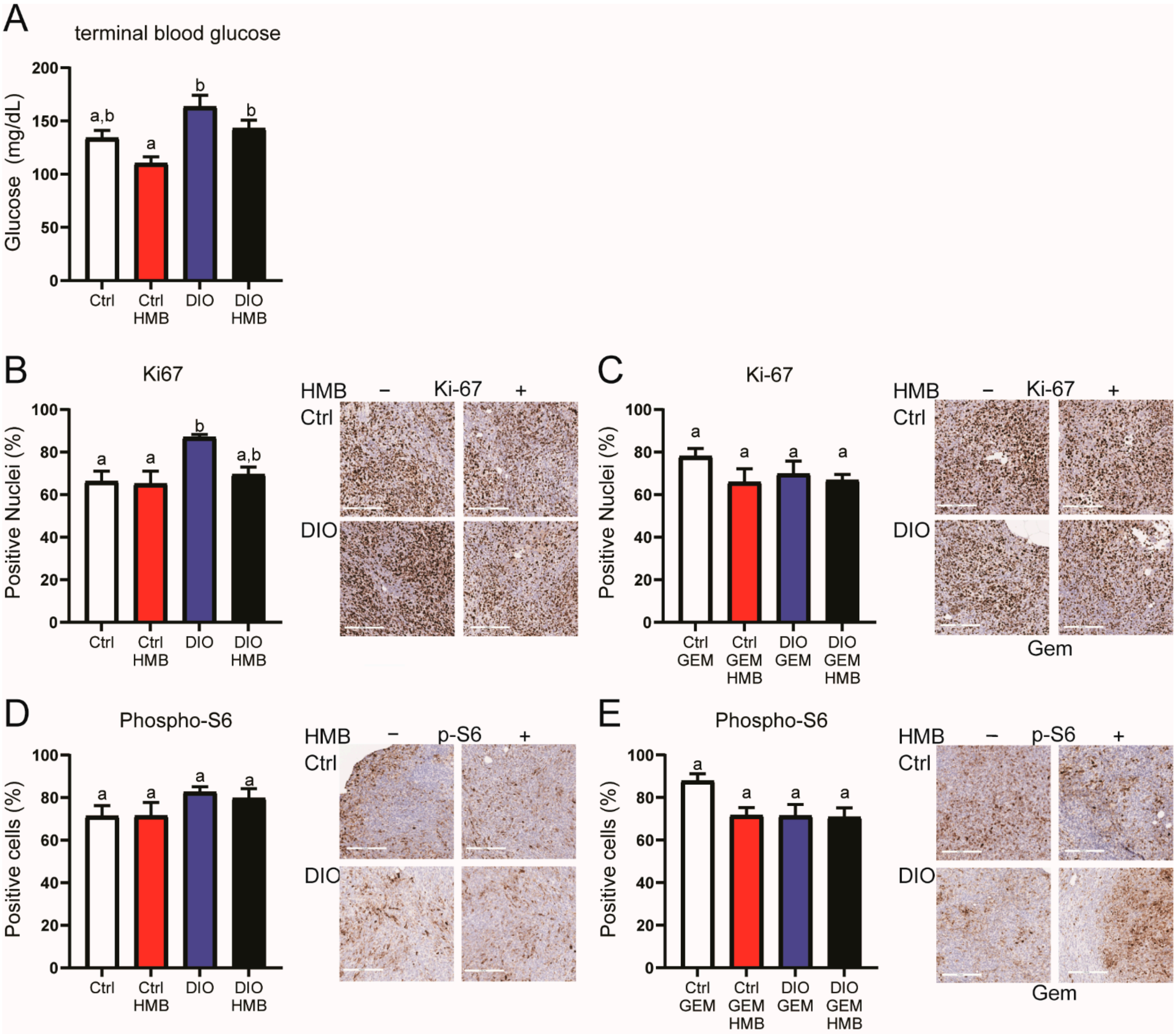

Figure S2. Blood glucose, proliferation, and apoptosis are not altered by HMB. (A) Fasted blood glucose following 6 hour fast (n=14/group). (B-E) immunohistochemistry staining of tumor sections for Ki67 (B-C) and phosphor-S6 (D-E), in treatment naïve (B&D) or gemcitabine treated tumors (C&E) (n=5-6/group). All data are presented as mean ± SEM. Differences are considered significant if $p < 0.05$ as indicated by different letters within the same graph.

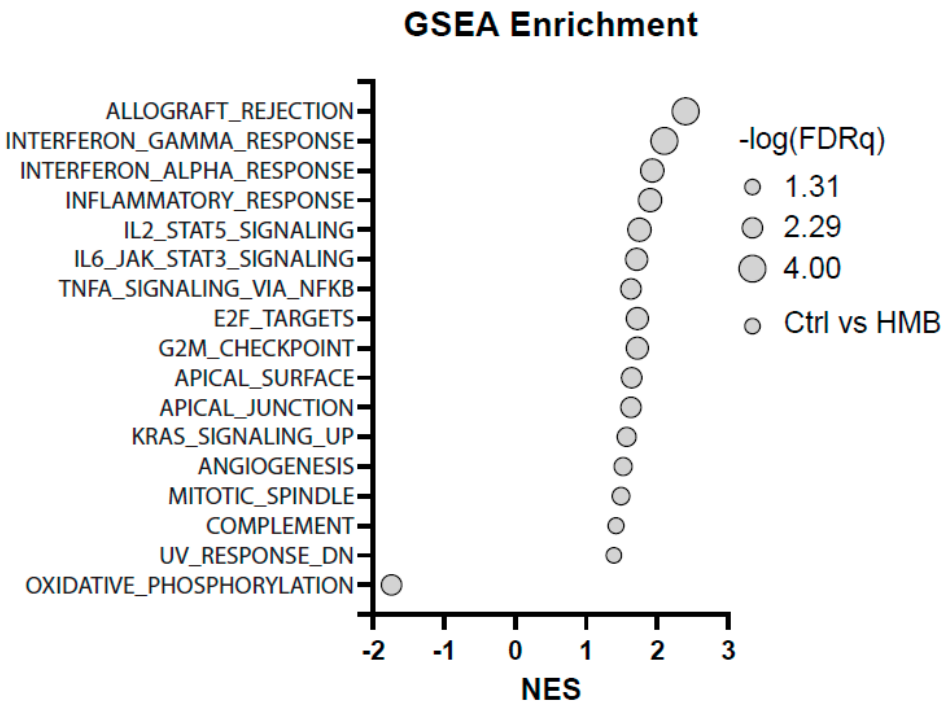

Figure S3. HMB supplementation alters GSEA Hallmark enrichments in muscles. Significant GSEA enrichments from Hallmark gene sets resulting from comparing Control vs HMB (grey), FDRq is denoted by bubble size (n=7/group).

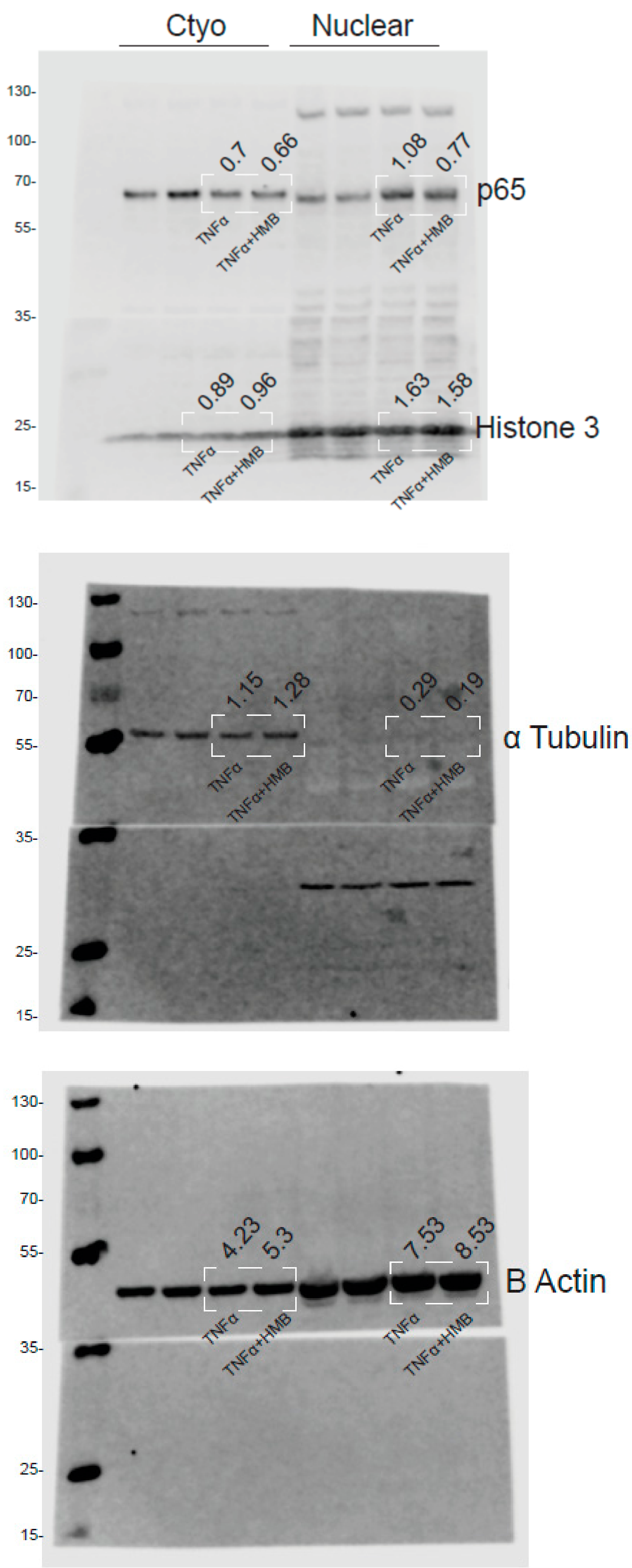

Figure S4. Uncropped Western blots for Figure 7E. C2C12 myotubes treated with HMB for 18 h in serum-free media containing 10 ng/ml TNFα. Western blot of nuclear p65 protein levels.
